# Bullatine A stimulates spinal microglial dynorphin A expression to produce anti-hypersensitivity in a variety of rat pain models

**DOI:** 10.1186/s12974-016-0696-2

**Published:** 2016-08-30

**Authors:** Qian Huang, Xiao-Fang Mao, Hai-Yun Wu, Teng-Fei Li, Ming-Li Sun, Hao Liu, Yong-Xiang Wang

**Affiliations:** King’s Lab, Shanghai Jiao Tong University School of Pharmacy, 800 Dongchuan Road, Shanghai, 200240 China

**Keywords:** Bullatine A, Anti-hypersensitivity, Spinal cord, Microglia, Dynorphin A, Pro-inflammatory cytokines

## Abstract

**Background:**

*Aconiti brachypodi* Radix (Xue-shang-yi-zhi-hao) has been prescribed to manage chronic pain, arthritis, and traumatic injuries. Bullatine A, a C_20_-diterpenoid alkaloid, is one of its principle effective compounds. This study aimed to investigate the anti-hypersensitivity of bullatine A in a variety of rat pain models and explore its mechanisms of action.

**Methods:**

Rat neuropathic pain, inflammatory pain, diabetic neuropathic pain, and bone cancer pain models were used. Dynorphin A and pro-inflammatory cytokines were measured in the spinal cord and cultured primary microglia. Double immunofluorescence staining of dynorphin A and glial and neuronal cellular markers was also measured in the spinal cord.

**Results:**

Subcutaneous and intrathecal injection of bullatine A dose-dependently attenuated spinal nerve ligation-, complete Freud’s adjuvant-, diabetes-, and bone cancer-induced mechanical allodynia and thermal hyperalgesia, with the efficacies of 45–70 % inhibition, and half-effective doses of 0.9–1.9 mg/kg for subcutaneous injection. However, bullatine A was not effective in blocking acute nociceptive response in the normal condition. Bullatine A specifically stimulated dynorphin A expression in microglia in the spinal cord in vivo and cultured primary microglia in vitro; the stimulatory effects were completely inhibited by the microglial inhibitor minocycline. In contrast, bullatine A did not have an inhibitory effect on peripheral nerve injury- or lipopolysaccharide-induced pro-inflammatory cytokine expression. The spinal anti-allodynic effects of bullatine A were entirely blocked by intrathecal injection of minocycline, the specific dynorphin A antiserum, and the selective k-opioid receptor antagonist.

**Conclusions:**

We, for the first time, demonstrate that bullatine A specifically attenuates pain hypersensitivity, regardless of the pain models employed. The results also suggest that stimulation of spinal microglial dynorphin A expression mediates bullatine A anti-nociception in pain hypersensitivity conditions.

## Background

**Fig. 1:**
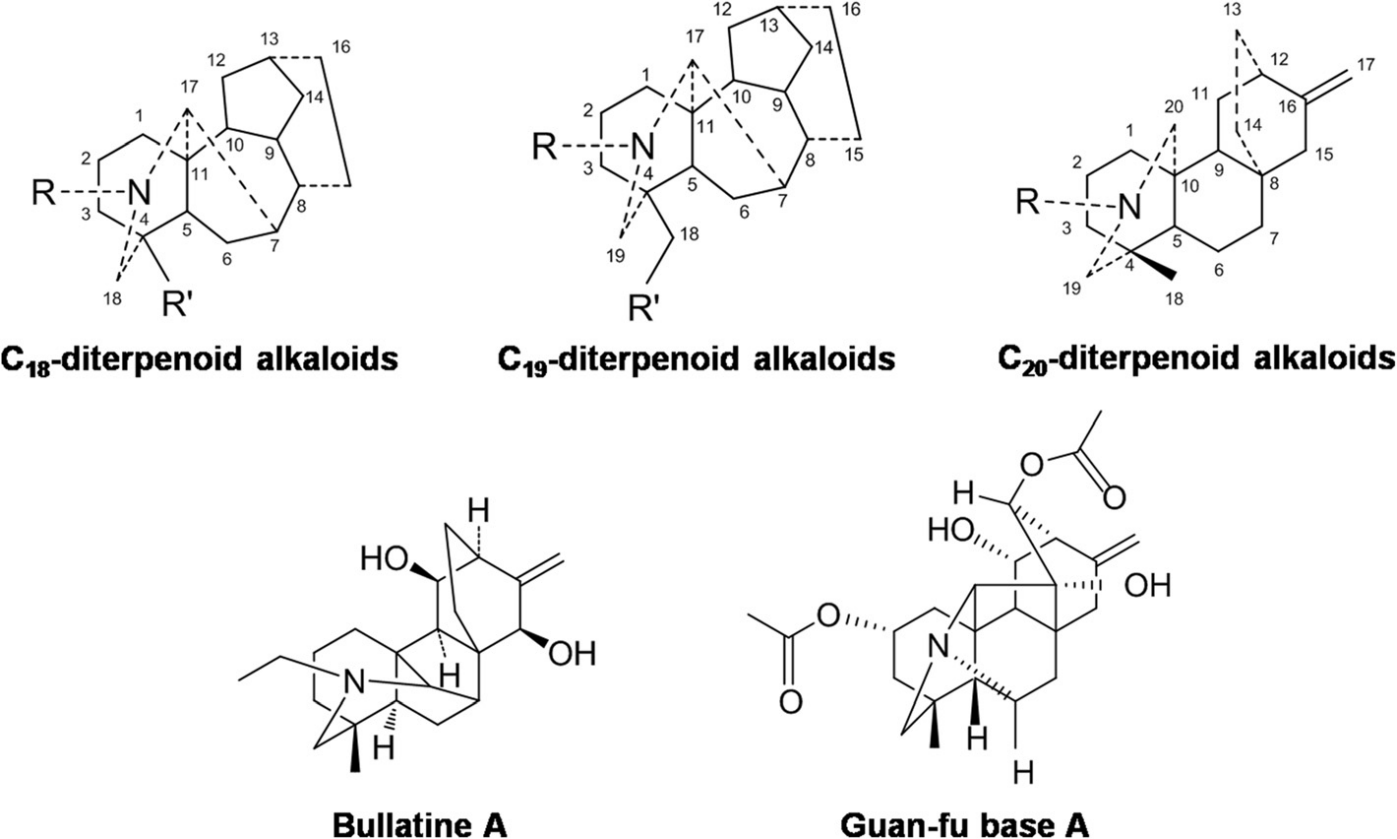
Chemical structures of C_18_-, C_19_-, and C_20_-diterpenoid alkaloids, bullatine A, and guan-fu base A

*Aconiti brachypodi* Radix (Xue-shang-yi-zhi-hao), the dried roots of *Aconitum brachypodum* Diels and several other morphologically similar species (genus *Aconitum*, family Ranunculaceae), is listed in the *Chinese Pharmacopoeia* for its analgesic and anti-rheumatic properties [1–3]. The bioactive extracts of *A. brachypodi* Radix, in the forms of pills, liniment, patch, and injection, are widely prescribed in China to manage chronic pain, arthritis, and traumatic injuries. As a principal group of compounds present in *Aconitum*, approximately 170 alkaloids have been identified and fell into four skeletal categories: C_18_-, C_19_-, C_20_- and *bis*-diterpenoid alkaloids [3–5]. Bullatine A, a C_20_-diterpenoid alkaloid, is one of the major effective and quality control ingredients identified from *A. brachypodi* Radix [6]. The chemical structures of C_18_-, C_19_-, and C_20_-diterpenoid alkaloids, as well as bullatine A, are presented in Fig. 1. Unlike the high toxicity of C_18_- and C_19_-diterpenoid alkaloids, such as aconitine, bulleyaconitine A, and lappaconitine, bullatine A exhibits significantly lower toxicity (oral half-lethal dose: bullatine A 754 mg/kg vs. aconitine 1.8 mg/kg in mice) [3, 7]. It was reported that systemic administration of bullatine A and the ethanol extract of *A. brachypodi* Radix including bullatine A effectively attenuated pain responses in the mouse hot-plate, acetic acid, and formalin tests [8]. However, no investigations have been published to date on the anti-nociceptive effects of bullatine A in pain hypersensitivity models.

The crucial role of spinal microglia has been recognized with regard to the initiation and development of chronic pain, including neuropathic pain, inflammatory pain, diabetic neuropathic pain, and bone cancer pain [9–13]. Activated microglia have been implicated in chronic pain states, leading to the production of pro-inflammatory cytokines, such as tumor necrosis factor-α (TNF-α), interleukin (IL)-6, and IL-1β [13, 14]. The cytokines released from activated microglia can consequently induce central sensitization of neurons in the spinal dorsal horn by altering the excitatory or inhibitory synaptic transmission, contributing to pain facilitation [15]. Li et al. showed that bullatine A, by selectively antagonizing P2X7 receptors, inhibited ATP-induced microglial death/apoptosis and P2X receptor-mediated inflammatory response [16]. On the other hand, bulleyaconitine A, a C_19_-diterpenoid alkaloid of *Aconitum*, was recently reported to exhibit potent anti-nociception through stimulation of the dynorphin A expression in spinal microglia [17].

In this paper, we aimed to investigate the anti-hypersensitive effects of bullatine A in a variety of rat models of pain hypersensitivity, including spinal nerve ligation-induced neuropathic pain, streptozotocin-induced diabetic neuropathic pain, complete Freud’s adjuvant (CFA)-induced inflammatory pain, and bone cancer pain. We also explored the mechanisms underlying bullatine A anti-nociception, particularly the involvement of expressions of spinal microglial dynorphin A and pro-inflammatory cytokines. In addition, guan-fu base A is an anti-arrhythmic C_20_-diterpenoid alkaloid [18–20] and has a similar carbon skeleton structure to bullatine A (Fig. 1). Its effects on nociception in neuropathy and microglial dynorphin A expression were also comparatively studied.

## Methods

### Drugs and reagents

Bullatine A and guan-fu base A were purchased from the National Institute for the Food and Drug Control (Beijing, China) with purity not less than 98 % determined by the manufacturer with high-performance liquid chromatography (HPLC). The molecular weights of both compounds were verified in house by a high-resolution mass spectrum (Waters Corporation, Milford, MA, USA). Dynorphin A(1–17) (sequence: YGGFLRRIRPKLKWDNQ) was synthesized by Dang Gang Peptides Co. (Hangzhou, China) with purity not less than 98 % determined by the manufacturer. Morphine hydrochloride, lidocaine, and minocycline were purchased from the Northeast Pharmaceuticals Group (Shenyang, China), the First Chengdu Pharmaceuticals Group (Chengdu, China), and Yuanye Biotech (Shanghai, China), respectively. 5′-Guanidinonaltrindole (GNTI) and streptozotocin were purchased from Sigma-Aldrich (St. Louis, MO, USA), and CTAP (sequence : FCYWRTXT) and naltrindole were purchased from Abcam (Cambridge, UK) and Tocris (Bristol, UK), respectively. The rabbit polyclonal antibody-neutralizing dynorphin A was purchased from Phoenix Pharmaceuticals (Burlingame, CA, USA). Based on the manufacturer’s information, the antiserum was specific to dynorphin A (100 %), but not to dynorphin B (0 %), β-endorphin (0 %), α-neo-endorphin (0 %), or leu-enkephalin (0 %). Its specificity was also validated by the antigen absorption test from other laboratories [21, 22]. All the drugs and reagents were dissolved or diluted in 0.9 % normal saline except streptozotocin which was freshly dissolved in the citrate buffer (pH 4.3).

### Experimental animals

Male and female adult (160–250-g body weight) and 1-day-old neonatal Wistar rats were obtained from the Shanghai Experimental Animal Institute for Biological Sciences (Shanghai, China). The adult animals were housed (three to four per cage) in the Shanghai Jiao Tong University Experimental Animal Center (Shanghai, China) at standard room temperature (22 ± 2 °C), under conditions of a 12/12-h reversed light-dark cycle (7:00 a.m.–7:00 p.m.), and received food and water ad libitum. Adult rats were accustomed to the laboratory environment for 3–5 days before the experiments. Experimental study groups (*n* = 6 in each group) were randomly assigned, and the researcher was blinded to the behavior tests. The research protocols were approved by the Animal Care and Welfare Committee of Shanghai Jiao Tong University and carried out in accordance with the animal care guidelines of the National Institutes of Health.

### Primary cell cultures

Glial cells and neurons were isolated from the cortex and spinal cord of 1-day-old neonatal rats. The isolated cortex and spinal cord were minced and then incubated with trypsin. Dissociated cells were suspended in Dulbecco’s modified Eagle’s medium (DMEM) supplemented with 10 % (vol/vol) fetal bovine serum (FBS), penicillin (100 U/mL), and streptomycin (100 μg/mL). For glial cell cultures, cell suspensions were plated in 75-cm^2^ tissue culture flasks (1 × 10^7^ cells/flask) pre-coated with poly-l-lysine and maintained at 37 °C in a 5 % carbon dioxide incubator. After culture for 8 days, microglial cells were prepared as floating cell suspensions by shaking the flasks at 260 rpm for 2 h. The aliquots were transferred to new plates, and unattached cells were removed by washing with serum-free DMEM. Harvested microglial cells exhibited a purity >95 %, as determined by the CD11b (OX42) or Iba-1 immunoreactivity. After culture for 11 days, astrocytes were prepared by shaking the flasks for 2 h followed by incubation with 10 mL of 0.05 % trypsin-ethylenediamine tetraacetic acid (Invitrogen, Grand Island, NY, USA) in a cell incubator for 15 min to separate the oligodendrocytes from the astrocytes. After trypsin neutralization with 10 mL of the complete DMEM medium, the floating cell suspensions were discarded. A nearly intact layer of astrocytes in the bed layer was then trypsinized and subcultured conventionally. Prepared astrocytes exhibited a purity >90 %, as determined by the glial fibrillary acidic protein (GFAP) immunoreactivity.

For the neuronal culture, cell suspensions were plated in plates pre-coated with poly-l-lysine (100 μg/mL). After 4 h of incubation in the DMEM with 10 % FBS, the medium was changed to the Neurobasal (Invitrogen, Grand Island, NY, USA) containing B27 supplement and 0.5 mM glutamine for further culture. Experiments were initiated 5–6 days after plating. Harvested neurons exhibited a purity >85 %, as determined by the NeuN immunoreactivity.

### RNA extraction, reverse transcription, and real-time quantitative polymerase chain reaction (PCR)

TRIzol reagent (Invitrogen, Grand Island, NY, USA) was used to isolate total RNA from rat ipsilateral spinal lumbar enlargements (L3–L5) and primarily cultured cells [23]. A sample of 1 μg of total RNA was reversely transcribed using a ReverTra Ace qPCR RT-Kit (Toyobo Co., Osaka, Japan). Real-time quantitative PCR was carried out with a Mastercycler ep realplex (Eppendorf, Germany) using the Realmaster Mix (SYBR Green I) (Toyobo Co., Osaka, Japan). The fold change was calculated using the 2^−ΔΔCt^ method after normalization to *gapdh*. The primers were as follows: 5′-CCA AGG TCA TCC ATG ACA AC-3′ (*gapdh* forward); 5′-TCA TCC ATG ACA AC-3′ (*gapdh* reverse) [24]; 5′-CCT GTC CTT GTG TTC CCT GT-3′ (prodynorphin forward); 5′-AGA GGC AGT CAG GGT GAG AA-3′ (prodynorphin reverse) [25]; 5′-CCC CGA CTA TGT GCT CCT CAC-3′ (TNF-α forward); 5′-AGG GCT CTT GAT GGC GGA-3′ (TNF-α reverse); 5′-GGA AGG CAG TGT CAC TCA TTG TG-3′ (IL-1β forward); 5′-GGT CCT CAT CCT GGA AGC TCC-3′ (IL-1β reverse); 5′-GGG ACT GAT GTT GTT GAC AGC C-3′ (IL-6 forward); and 5′-CAT ATG TAA TTA AGC CTC CGA CTT GTG-3′ (IL-6 reverse) [26].

For the ex vivo study, sham and neuropathic rats received two intrathecal treatments: (1) 10 μl saline + 10 μl saline; (2) 100 μg minocycline + 10 μl saline; (3) 10 μl saline + 10 μg bullatine A; and (4) 100 μg minocycline + 10 μg bullatine A. The second treatment was administered 4 h after the first treatment, and the ipsilateral spinal lumbar enlargements were obtained 1 h later. The expressions of prodynorphin A, TNF-α, IL-1β, and IL-6 were measured using the real-time PCR.

For the in vitro study, cultured primary cells were under two treatments in the presence and absence of lipopolysaccharides (LPS, 10 ng): (1) control + control; (2) 60 μM minocycline + control; (3) control + 10 μM bullatine A; and (4) 60 μM minocycline + 10 μM bullatine A. The concentration of minocycline was based on the previous references [26–29]. The second treatment was administered 1 h after the first treatment, and the microglia were collected 6 h later. The expressions of prodynorphin A, TNF-α, IL-1β, and IL-6 were measured using the real-time PCR.

### Intrathecal catheterization and injection in rats

An 18-cm polyethylene catheter (PE-10: 0.28-mm inner diameter and 0.61-mm outer diameter; Clay Adams, Parsippany, NJ, USA) with a volume of 13 μL was inserted into the rat lumbar level of the spinal cord under inhaled isoflurane anesthesia (4 % for induction and 1 % for maintenance) run by an anesthesiameter (Ugo Basile Gas Anesthesia System, Comerio, Italy). Two days after recovery from anesthesia, the correct positioning of the catheter in the spinal cord was verified by administering 4 % lidocaine (10 μL followed by 15 μL of saline for flushing) with a 50-μL microinjector (Shanghai Anting Microinjector Factory, Shanghai, China). Only rats that had no motor impairment following insertion of the intrathecal catheter were considered for the study, and only rats that developed immediate bilateral paralysis of the hindlimbs following intrathecal administration of lidocaine were selected for the study.

### Rat model of neuropathic pain

To induce neuropathic pain, the adult male rats were subjected to spinal nerve ligation as described previously [23, 30]. Under inhaled isoflurane anesthesia, the left L5 and L6 spinal nerves were isolated and tightly ligated with 6–0 silk thread. Sham rats were under the same procedure except for the spinal nerves were not ligated. After ligation, the wound was sutured and the rats were allowed to recover. Of the spinal nerve-ligated animals, only those with marked unilateral allodynia to mechanical stimulation (hindlimb withdrawal thresholds in the operated side <8 g), and no major impairment were included in the study. When intrathecal injection was needed, the intrathecal catheterization was performed in rats at the same time just before spinal nerve ligation. Drug testing started on 2–4 weeks after the surgery of spinal nerve ligation.

### Rat model of complete Freud’s adjuvant (CFA)-induced inflammatory nociception

CFA inflammatory nociception was induced according to Butler et al. [31] and Fan et al. [26]. Briefly, 100 μL of CFA (Sigma-Aldrich, MO, USA) was injected into the tibiotarsal joint of the left hindpaw of each adult male rat under mild inhaled isoflurane anesthesia. The tibiotarsal joint injection of CFA produced immediate and long-lasting inflammation and pain hypersensitivity. The rats entered into the behavior tests 2 days after CFA injection.

### Rat model of diabetic neuropathic pain

The adult male rats were fasted for 16 h before receiving a single intravenous injection of streptozotocin (40 mg/kg) [24, 32]. High blood sugar levels were observed on the third day after injection. Mechanical allodynia was measured by electronic von Frey hairs 4–5 weeks afterward.

### Rat model of bone cancer pain

The rat bone cancer pain model was produced as described previously [23, 33]. Adult, female rats were anesthetized with intraperitoneal pentobarbital (50 mg/kg). Incisions were made along the patellar ligament to expose the head of the left tibia with minimal damage. A 23-gauge needle was inserted at the site of intercondylar eminence and pierced 7 mm below the knee joint into the medullary cavity of the tibia. The needle was then removed and attached to a 10-μL microinjection syringe. Walker 256 carcinoma cells (4 × 10^5^) in 10 μL the phosphate buffer solution were slowly injected into the tibia cavity. The syringe remained for an additional 1 min to prevent the carcinoma cells from leaking out. The injection site was then closed with aseptic bone wax, and the wound was then closed and dusted with penicillin powder. After recovery from the inoculation surgery, rats were returned to their home cages. The withdrawal thresholds of contralateral and ipsilateral hindlimbs were measured by the application of electronic von Frey hairs 2 weeks afterward.

### Behavioral assessments of mechanical allodynia and thermal hyperalgesia in rats

To evaluate mechanical allodynia, the animals were acclimatized for at least 30 min to the test environment, namely a plexiglass box on a metal grid. The hindpaw withdrawal threshold was measured by a 2391 CE Electronic von Frey hair (IITC Life Science Inc, Woodland Hill, CA, USA). The monofilament (with forces ranging from 0.1 to 90 g) was applied to the foot pad with increasing force until the rats suddenly withdrew their hindlimbs. The lowest force producing a withdrawal response was considered as the threshold. Three repeated measurements were made at intervals of approximately 3 min, and the three threshold values were averaged for each hindpaw at each time point.

To assess heat hyperalgesia, rats were put in a plexiglass box on an elevated glass surface. Following an adaption period of at least 30 min, radiant heat source (at a low density of 45) was applied to the plantar medial surface of each hindpaw. The hindpaw withdrawal latency was measured by a 390G Plantar Test Analgesia Meter (IITC Life Science Inc.). To prevent tissue damage, the cutoff was set at 30 s. The paw withdrawal latency was defined as the time from the onset of radiant heat application to the withdrawal of the hindpaws. Both hindpaws were tested independently three times with a 5-min interval between the trials. The result was calculated as the mean of the three repeated measurements.

For the dose response of systemic bullatine A study, two groups of rats from four different pain models were subcutaneously injected with 1 mL/kg saline or 0.3, 0.7, 2, 7, and 20 mg/kg of bullatine A sequentially in 1-h intervals to yield a cumulative dose of approximately 0.3, 1, 3, 10, and 30 mg/kg. The mechanical or thermal thresholds were measured 1 h after each injection. For the dose response of intrathecal bullatine A study, six groups of neuropathic rats received a single intrathecal injection of saline (10 μL) and four doses of bullatine A (0.3, 1, 3, 10 and 30 μg). The withdrawal thresholds to mechanical and thermal stimuli in both contralateral and ipsilateral hindlimbs were measured prior to and 0.5, 1, 2, and 4 h after injection.

For the antagonist intervention study, groups of neuropathic rats in each study received two intrathecal injections of the vehicle (10 μL) + bullatine A (10 μg)/dynorphin A (1 μg) and the antagonist + bullatine A (10 μg)/dynorphin A (1 μg). All of the second treatments were administered 0.5 h after the first treatment except minocycline which was given 4 h after the first injection. The withdrawal thresholds to mechanical stimuli in both contralateral and ipsilateral hindlimbs were measured prior to and 0.5, 1, 2 and 4 h after the second injection. The time and dose regimens of the antagonists in the studies were based on the following references: (1) the microglial inhibitor minocycline (100 μg) [34, 35]; (2) the specific dynorphin A antiserum (1:10 dilution) [17]; and (3) the selective μ-opioid antagonist CTAP (10 μg), k-opioid antagonist GNTI (50 μg), and σ-opioid antagonist naltrindole (5 μg) [36].

### Immunofluorescence staining

Double immunofluorescence labeling of dynorphin A and microglia, astrocytes, or neurons on spinal cord sections was undertaken and observed using a TCS SP8 confocal microscope (Leica Microsystems, Wetzlar, Germany) by modified protocols [24]. Rats were anesthetized by pentobarbital injection (40 mg/kg) and intracardially perfused with 100 mL normal saline, followed by 60 mL of 4 % paraformaldehyde (*w*/*v*) in PBS. Spinal lumbar enlargements (L3–L5) were collected and fixed in 4 % buffered paraformaldehyde for 12 h and placed in 30 % sucrose solution for 48 h at 4 °C. Tissues were entrapped in the OCT embedding agent (Leica Microsystems) and cut into 30-μm-thick frozen sections, which were incubated in 10 % goat serum (*v*/*v*) and 0.5 % Triton X-100 (*v*/*v*) in PBS for 1 h, then incubated with the rat dynorphin A antibody (1:100; rabbit polyclonal, Phoenix Pharmaceuticals) and other primary antibodies at 4 °C for 24 h. Spinal neuronal and glial cells were identified by the following cellular markers: Iba-1 (1:100; mouse monoclonal; Millipore, Darmstadt, Germany) for microglia, GFAP (1:100; mouse polyclonal; Millipore) for astrocytes, and NeuN (1:60; mouse polyclonal; Millipore) for neurons. The dynorphin A was visualized with the Alexa-555-conjugated goat anti-rabbit secondary antibody (1:200, Invitrogen). Other antibodies were detected with the Alexa-488-conjugated goat anti-mouse secondary antibody (1:200, Invitrogen).

For quantity measurement of dynorphin A and/or Iba-1/GFAP/NeuN-immunopositive cell intensity in the spinal cord, photomicrographs of the medial three fourths of the superficial dorsal horn (laminas I–V) were captured under a 10× or 40× magnification. The positively stained surface area was measured by a researcher blinded to the experimental conditions using a computer-assisted image analysis program (ImageJ Software, National Institutes of Health) after low and high thresholds were set to exclude background fluorescence and include immunofluorescent intensity measurements only from positively stained areas. A co-localization analysis was performed using ImageJ software with a co-localization finder to generate images in which co-localized pixels appeared as white. The same configuration setup was used to measure all surface areas in each experimental group at the same time. The averaged percentage immunolabeled surface area was the fraction of the positive immunofluorescent surface area of the total measured picture area from three nonadjacent sections of each spinal cord. Data were then calculated from six rats of each group.

### Data evaluation and statistical analysis

The percentage of the maximal possible effect (% MPE) was calculated using the following formula: (post-drug threshold in ipsilateral hindlimb − baseline threshold in ipsilateral hindlimb)/(baseline threshold in contralateral hindlimb − baseline threshold in ipsilateral hindlimb) × 100 [37]. The % MPE values near 100 indicate normal mechanical thresholds (i.e., near contralateral thresholds) while values near 0 indicate allodynia. For the dose–response curve analysis, the parameters, i.e., minimum effect, maximum effect (*E*_max_), half-effective dose (ED_50_) or half-effective concentration (EC_50_), and Hill coefficient (*n*), were calculated by fitting non-linear least-square curves to the relation *Y* = *a* + *bx*, where *x* = [*D*]^*n*^/(ED_50_^*n*^ + [*D*]^*n*^) or [*C*]^*n*^/(EC_50_^*n*^ + [*C*]^*n*^). The value of ED_50_ or EC_50_ and *b* (*E*_max_) were projected by yielding a minimum residual sum of squares of deviations from the theoretical curve [38]. The data were expressed as means ± SEM or with 95 % confidence limits, and there were no missing data. The statistical significance was evaluated by unpaired and two-tailed Student *t* tests and one-way or two-way repeated-measure analysis of variance (ANOVA) in Prism (version 5.01, GraphPad Software, Inc., San Diego, CA, USA). The post hoc Student–Newman–Keuls test was conducted when the effect of the drug (dose) (for the one-way ANOVA, the factor was drug [dose]; for the two-way ANOVA, the factors were drug [dose], time, and their interaction) was observed to be statistically significant. The probability values were two-tailed, and the statistical significance criterion *P* value was 0.05.

## Results

### Systemic bullatine A specifically attenuated pain hypersensitivity in a variety of pain models

**Fig. 2:**
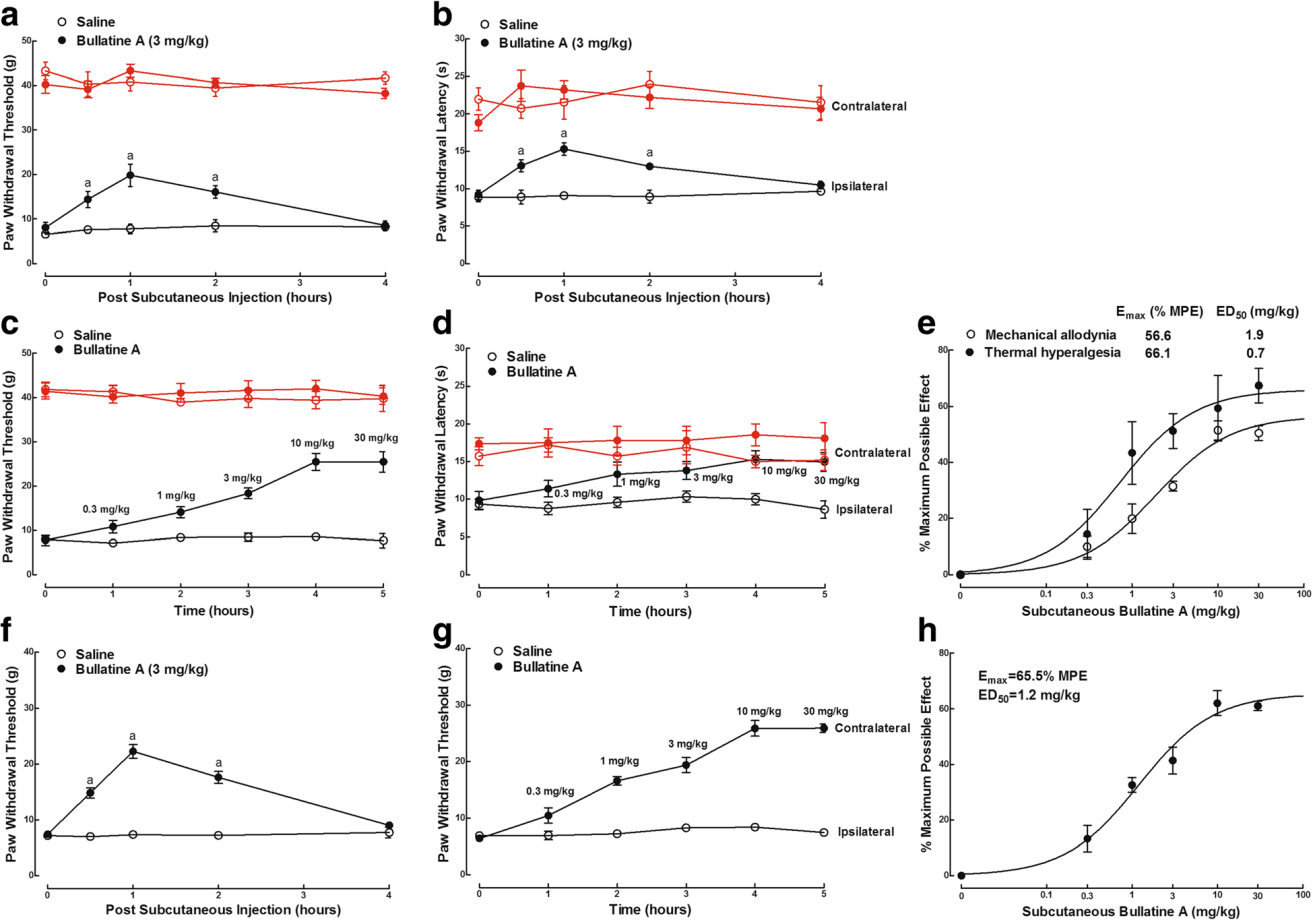
Inhibitory effects of bullatine A administered subcutaneously, in a single dose (3 mg/kg) or the cumulative dose range of 0.3 to 30 mg/kg, on mechanical allodynia and heat hyperalgesia in neuropathic (**a**–**e**) and diabetic (**f**–**h**) rats. Neuropathic pain was induced by tight ligation of L5/L6 spinal nerves, and diabetic neuropathic pain was induced by intravenous injection of streptozotocin (40 mg/kg). **e**, **h** Dose–response analysis of bullatine A on % maximum possible effect (% MPE) at 1 h after injection was best projected by the non-linear least-squares method. The data are presented as means ± SEM (*n* = 6 in each group). Symbol *a* denotes statistical significance compared with the saline control group (*P* < 0.05, two-way ANOVA followed by the post hoc Student–Newman–Keuls tests)

The anti-nociceptive effect of bullatine A was first examined in the rat model of neuropathic pain. Tight ligation of peripheral L5/L6 spinal nerves induced marked ipsilateral mechanical allodynia and thermal hyperalgesia. Two groups of neuropathic rats received subcutaneously injections of the vehicle (0.9 % saline, 1 mL/kg) and bullatine A (3 mg/kg). The paw withdrawal responses to mechanical and thermal stimuli were measured before and 0.5, 1, 2, and 4 h after injection. Subcutaneous bullatine A time-dependently alleviated mechanical allodynia (Fig. 2a) and thermal hyperalgesia (Fig. 2b) in ipsilateral paws, with the peaks at 1 h and durations of nearly 4 h (*P* < 0.05, by two-way ANOVA followed by the post hoc Student–Newman–Keuls tests). Subcutaneous bullatine A did not significantly alter normal withdrawal response to mechanical and heat stimuli in contralateral paws. No apparent sedation or motor side effects of bullatine A were observed during the study period. In addition, cumulative dose–response of bullatine A was conducted in two groups of neuropathic rats. Animals received repetitive subcutaneous injection of saline or bullatine A at 1-h intervals. The mechanical or thermal thresholds were measured before and 1 h after each dose injection. Subcutaneous bullatine A dose-dependently alleviated mechanical allodynia (Fig. 2c) and thermal hyperalgesia (Fig. 2d) in ipsilateral paws. The dose–response analyses were projected from the % MPE values from each dose for the blockade of mechanical allodynia and heat hyperalgesia. The ED_50_ values calculated were 1.9 and 0.7 mg/kg, and the *E*_max_ values were 56.6 and 66.1 % MPE, respectively (Fig. 2e).

Intravenous injection of streptozotocin (40 mg/kg) in rats produced immediate and permanent high blood sugar (>16.7 mmol/L) and resulted in stable painful neuropathy 4–5 weeks later, represented by bilateral mechanical allodynia [39]. Subcutaneous injection of bullatine A (3 mg/kg) was effective in attenuation of mechanical allodynia in a time-dependent manner, with the peak effect at 1 h after injection (*P* < 0.05, by two-way ANOVA followed by the post hoc Student–Newman–Keuls tests) (Fig. 2f). Cumulative doses (0.3–30 mg/kg) of subcutaneous bullatine A attenuated mechanical allodynia in a dose-dependent manner (Fig. 2g), with the ED_50_ value of 1.2 mg/kg and *E*_max_ value of 65.5 % MPE, which were projected from the dose–response analyses using % MPE at 1 h after injection calculating from the pre-streptozotocin paw withdrawal thresholds of 37.9 ± 1.9 g (Fig. 2h).

**Fig. 3:**
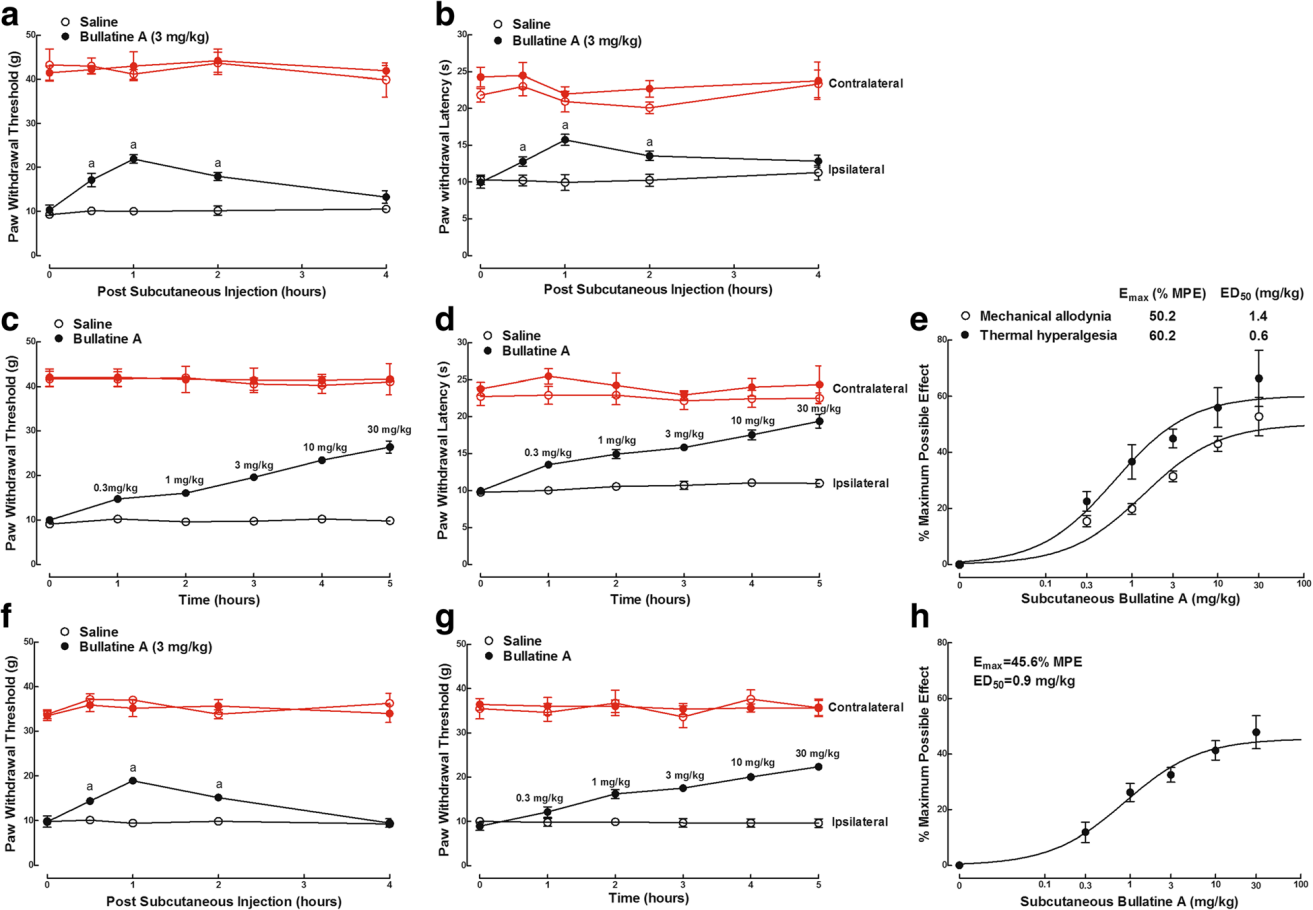
Inhibitory effects of bullatine A administered subcutaneously, in a single dose (3 mg/kg) or the cumulative dose range of 0.3 to 30 mg/kg, on mechanical allodynia and heat hyperalgesia in complete Freud’s adjuvant (CFA) induced inflammatory pain (**a**–**e**) and bone cancer pain (**f**–**h**) rats. The inflammatory model was induced by tibiotarsal joint injection of CFA (100 μL), and the bone cancer pain model was induced by tibia implantation of Walker 256 carcinoma cells. **e**, **h** Dose–response analysis of bullatine A on % maximum possible effect (% MPE) at 1 h after injection was best projected by the non-linear least-squares method. The data are presented as means ± SEM (*n* = 6 in each group). Symbol *a* denotes statistical significance compared with the saline control group (*P* < 0.05, two-way ANOVA followed by the post hoc Student–Newman–Keuls tests)

We further determined the anti-nociceptive effect of bullatine A in CFA-induced acute inflammatory nociception. Two groups of CFA-treated rats received subcutaneous injection of the vehicle (0.9 % saline, 1 mL/kg) and bullatine A (3 mg/kg). Subcutaneous injection of bullatine A did not significantly alter the paw withdrawal responses in contralateral paws during the 4-h observation period. However, bullatine A significantly blocked mechanical allodynia (Fig. 3a) and thermal hyperalgesia (Fig. 3b) in ipsilateral paws in a time-dependent manner, with the peak of 1 h after injection (*P* < 0.05, by two-way ANOVA followed by the post hoc Student–Newman–Keuls tests). In a cumulative dose range of 0.3 to 30 mg/kg, subcutaneous injection of bullatine A dose-dependently alleviated mechanical allodynia (Fig. 3c) and thermal hyperalgesia (Fig. 3d) in ipsilateral paws. The calculated ED_50_ values for the blockade of mechanical allodynia and thermal hyperalgesia were 1.4 and 0.6 mg/kg, and the *E*_max_ values were 50.2 % MPE and 60.2 % MPE, respectively (Fig. 3e).

Tibia implantation of cancer cells produced progressive mechanical allodynia in rats 14 days after surgery. As shown in Fig. 3f, subcutaneous injection of bullatine A (3 mg/kg) blocked bone cancer-induced mechanical allodynia in ipsilateral paws with the peak effect at 1 h and the duration of almost 4 h (*P* < 0.05, by two-way ANOVA followed by the post hoc Student–Newman–Keuls tests). It did not significantly affect withdrawal thresholds in contralateral paws. Subcutaneous injection of bullatine A (0.3 to 30 mg/kg, cumulative doses) dose-dependently increased withdrawal thresholds in ipsilateral paws (Fig. 3g), with the ED_50_ value of 0.9 mg/kg and *E*_max_ value of 45.6 % MPE calculated from the % MPE values at 1 h after injection (Fig. 3h).

### Intrathecal bullatine A specifically attenuated neuropathic pain

**Fig. 4:**
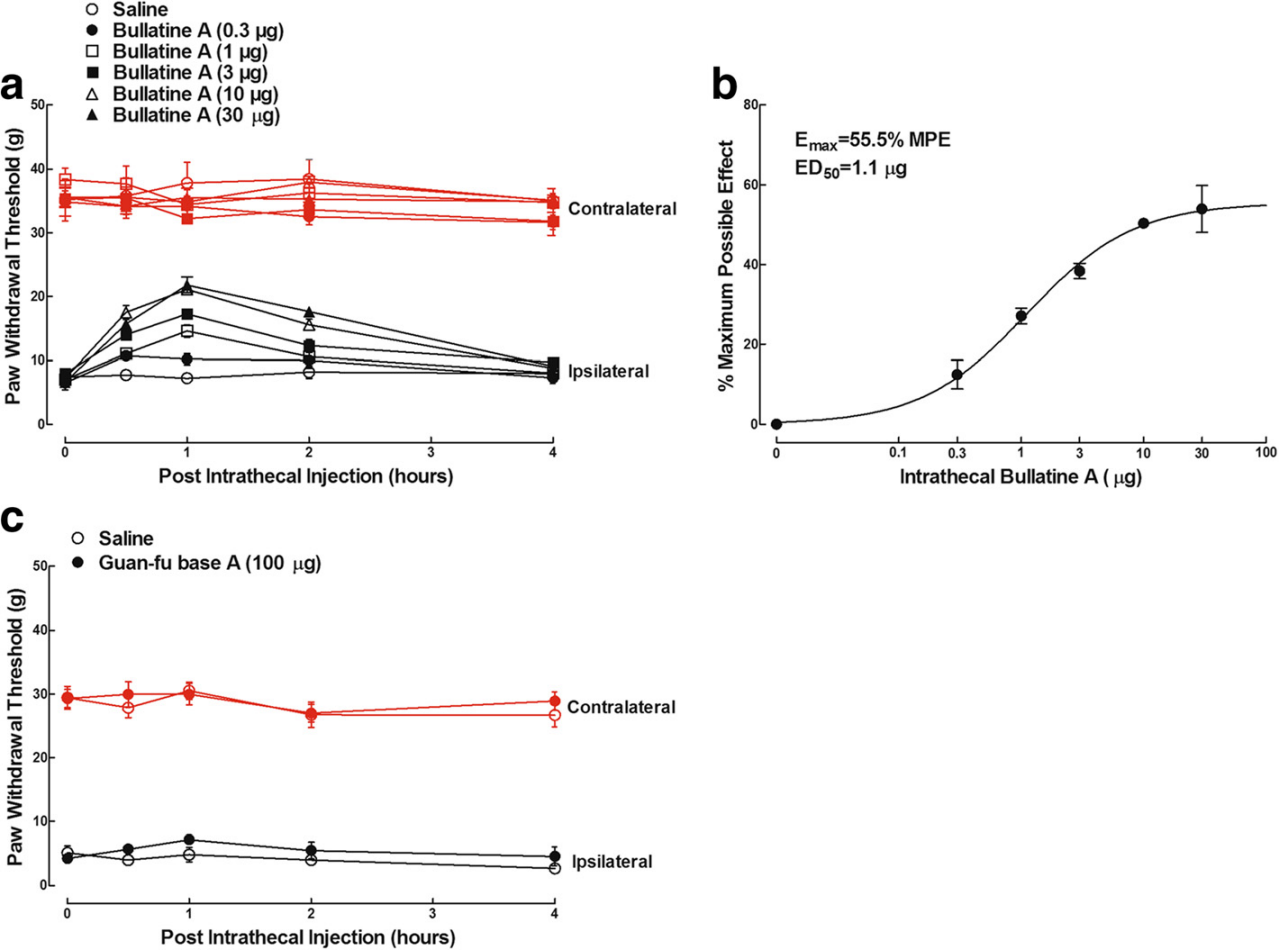
Effects of bullatine A (**a**, **b**) and guan-fu base A (**c**) administered intrathecally on mechanical allodynia in neuropathic rats. Neuropathic pain was induced by tight ligation of L5/L6 spinal nerves. **b** Dose–response analysis of bullatine A on % maximum possible effect (% MPE) at 1 h after injection was best projected by the non-linear least-squares method. The data are presented as means ± SEM (*n* = 6 in each group)

In order to test the anti-nociceptive efficacy of bullatine A in the spinal cord, six groups of neuropathic rats received a single intrathecal injection of saline (10 μL) or bullatine A at different doses (0.3, 1, 3, 10, and 30 μg). As shown in Fig. 4a, intrathecal bullatine A markedly suppressed mechanical allodynia in ipsilateral paws in a dose-dependent manner but did not significantly alter withdrawal thresholds in contralateral paws. No apparent sedation or motor side effects of bullatine A were observed during the study period. The anti-allodynic effect was time-dependent, with a peak effect at 1 h after injection and a duration of 4 h. Dose–response analysis showed that the ED_50_ value was 1.1 μg and *E*_max_ value was 55.5 % MPE, as calculated from the 1 h data after injection (Fig. 4b). On the contrary, intrathecal injection of the bullatine A analog guan-fu base A, at the most tolerable dose of 100 μg, did not show any significant anti-allodynic effects (Fig. 4c).

### Bullatine A upregulated the spinal microglial prodynorphin expression

**Fig. 5:**
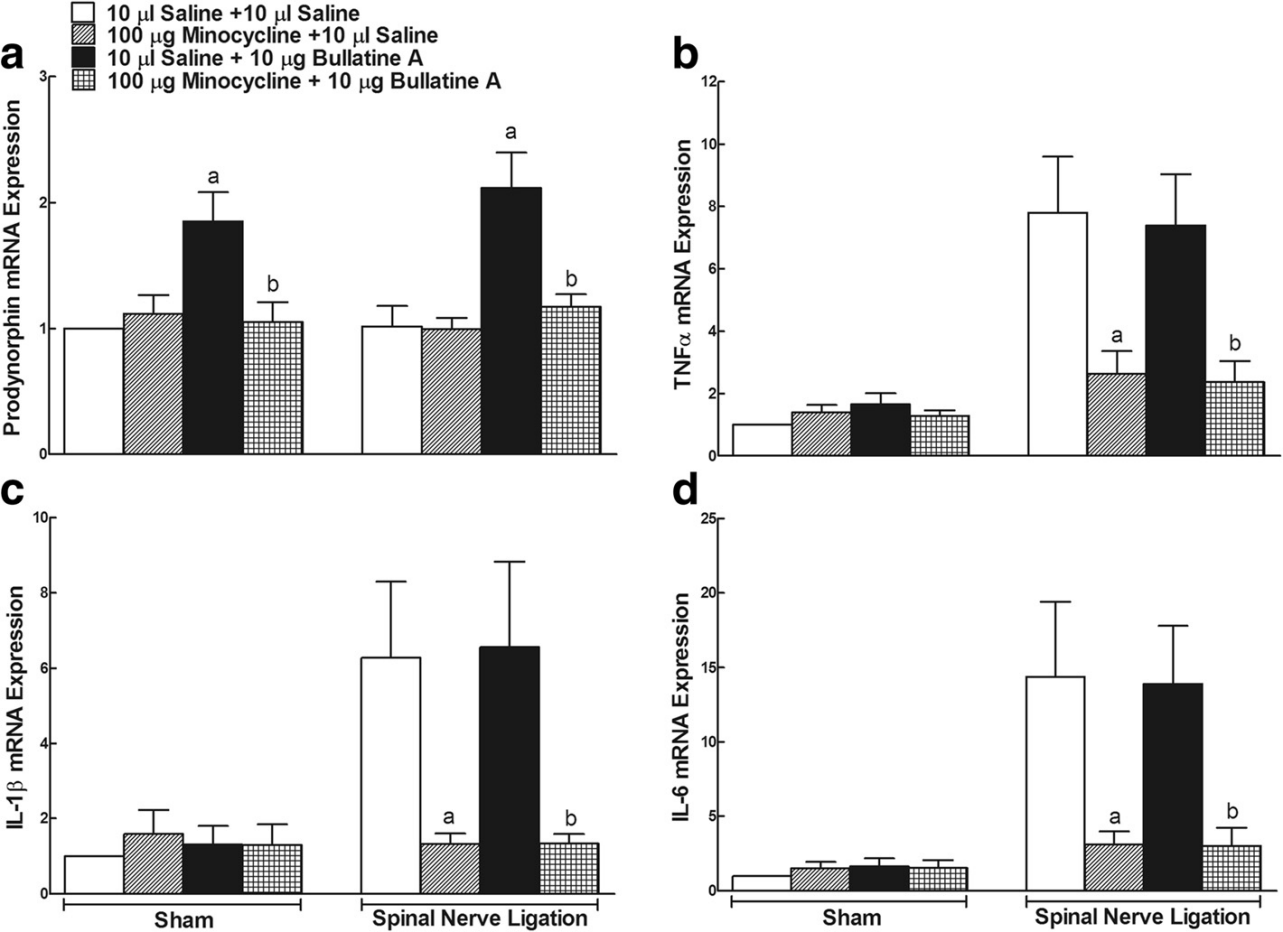
Effects of the intrathecal injection of bullatine A on the gene expression of prodynorphin (**a**) and pro-inflammatory cytokines, including tumor necrosis factor (TNF)-α (**b**), interleukin (IL)-1β (**c**), and IL-6 (**d**) in sham rats and neuropathic rats. Peripheral neuropathy was induced by the tight ligation of L5/L6 spinal nerves, and sham rats were under the same procedure except for the spinal nerves which were not ligated. The ipsilateral spinal lumbar enlargements were obtained 1 h after the intrathecal injection of saline or bullatine A (10 μg). The expression of prodynorphin and pro-inflammatory cytokines was measured by real-time quantitative PCR relative to the gapdh gene. The data are presented as means ± SEM (*n* = 6 in each group). Symbols *a* and *b* denote statistical difference compared with the saline plus saline group and saline plus bullatine A group in sham or neuropathic rats (*P* < 0.05, one-way ANOVA followed by the post hoc Student–Newman–Keuls test)

To test whether bullatine A affected the spinal gene expressions of dynorphin A and pro-inflammatory cytokines, four groups of neuropathic rats received intrathecal injection of saline (10 μL) or minocycline (100 μg) followed by a single dose of saline (10 μL) or bullatine A (10 μg) 4 h afterward. The rats were sacrificed 1 h after the second treatment, and ipsilateral lumbar enlargements (L3–L5) of the spinal cord were obtained. The cellular prodynorphin gene expression relative to gapdh was measured by real-time quantitative PCR in spinal homogenates. Since we recently reported that the C_19_-diterpenoid alkaloid bulleyaconitine-increased dynorphin A level measured by enzyme-linked immunosorbent assay was in parallel with the prodynorphin gene expression [17], the prodynorphin gene expression was only measured in this study. As shown in Fig. 5a, peripheral nerve injury approximately 20 days after surgery did not significantly change the basal prodynorphin gene expression, which was consistent with our previous results in which spinal nerve injury did not alter the basal spinal dynorphin A level [17]. Intrathecal bullatine A significantly increased the prodynorphin expression by 1.9- and 2.1-fold in the sham and neuropathic rats, respectively. Intrathecal injection of the microglial inhibitor minocycline [40, 41] completely inhibited bullatine A-increased prodynorphin expression (*P* < 0.05, by one-way ANOVA followed by the post hoc Student–Newman–Keuls tests), although it did not significantly alter the baseline prodynorphin expression.

The expression of pro-inflammatory cytokines was also measured in the same rats. Compared to the sham rats, spinal nerve ligation dramatically increased the spinal expression of TNF-α, IL-1β, and IL-6 by approximately 8-, 6- and 14-fold, respectively (*P* < 0.05, by one-way ANOVA followed by post hoc Student–Newman–Keuls tests). Pretreatment with minocycline completely blocked nerve injury-increased TNF-α (Fig. 5b), IL-1β (Fig. 5c), and IL-6 (Fig. 5d) expression (*P* < 0.05, by one-way ANOVA followed by post hoc Student–Newman–Keuls tests). In contrast, intrathecal administration of bullatine A did not significantly affect the gene expression of cytokines, including TNF-α (Fig. 5b), IL-1β (Fig. 5c), and IL-6 (Fig. 5d), in either sham or neuropathic rats.

**Fig. 6:**
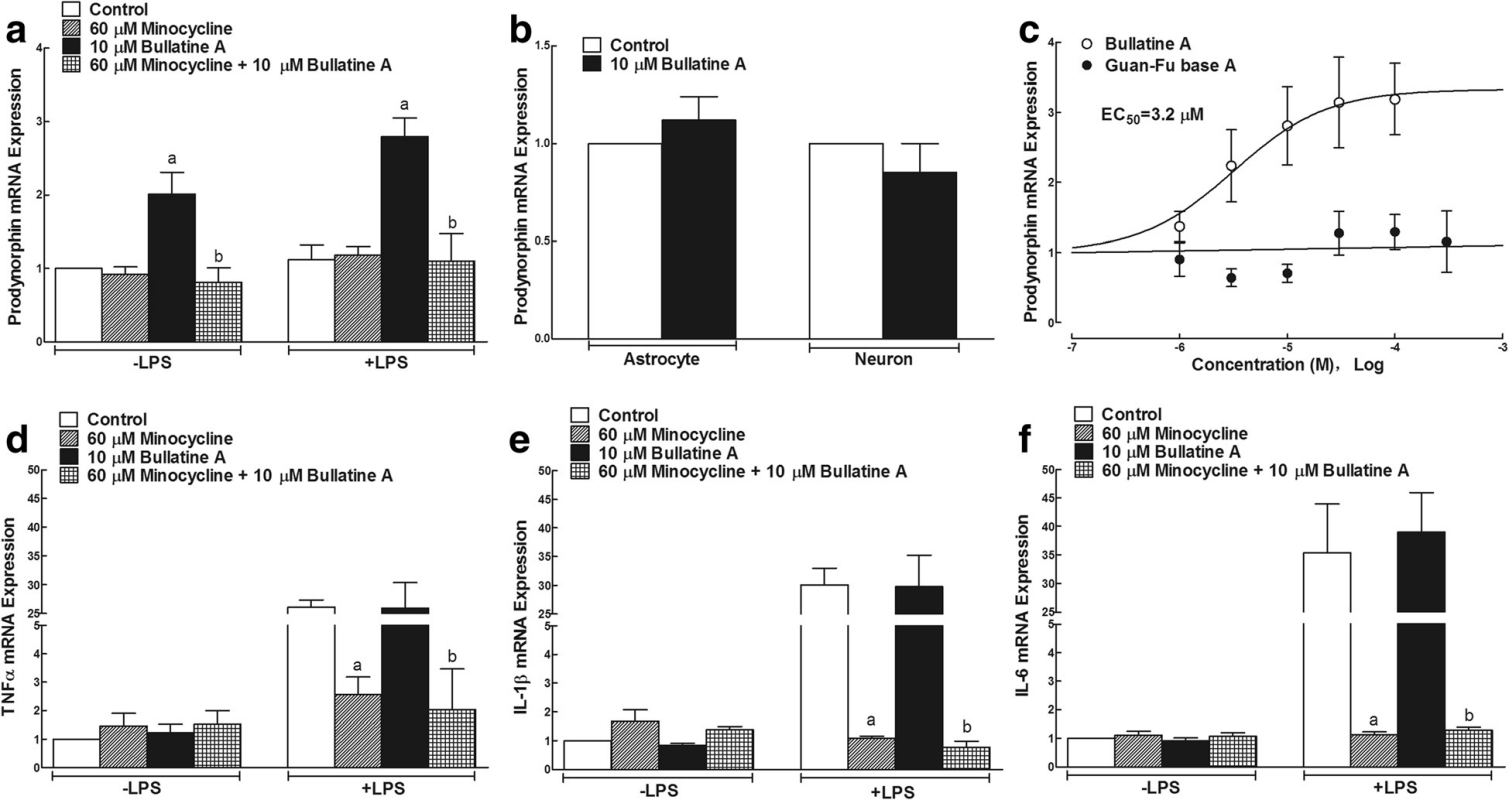
Effects of bullatine A and guan-fu base A on gene expressions of prodynorphin (**a**, **b**, **c**) and the tumor necrosis factor (TNF)-α (**d**), interleukin (IL)-1β (**e**), and IL-6 (**f**) in primary cultures of microglia, astrocytes, and neurons in the presence and absence of lipopolysaccharides (LPS). The primary microglial cells, astrocytes, and neurons were derived from the cortex of neonatal rats. Minocycline (60 μM) was treated 1 h before LPS (10 ng/mL) and bullatine A (10 μM) treatment. The cultured cells were collected 6 h later, and the expression of prodynorphin and pro-inflammatory cytokines was determined by real-time quantitative PCR relative to the gene of gapdh. The data are presented as means ± SEM (*n* = 3–5 in each treatment, with three independent repeats). Symbols *a* and *b* denote statistical difference compared with the control group and bullatine A group in the presence and absence of LPS (*P* < 0.05, one-way ANOVA followed by the post hoc Student–Newman–Keuls test)

We further determined the effects of bullatine A on the expression of prodynorphin and pro-inflammatory cytokines in microglial cells in the presence and absence of LPS. Cultured primary microglial cells (1 × 10^6^ cells per well) from the cortex of neonatal rats were treated with minocycline (60 μM) 1 h prior to LPS (10 ng/mL) or bullatine A (10 μM) treatment, and microglial cells were collected 6 h later. Treatment with minocycline at 60 μM for 24 h did not affect microglial viability measured by the MTT assay (100 % ± 9.5 % vs. 103.8 % ± 6.0 %, *n* = 5), consistent with the previous report. As shown in Fig. 6a, LPS (10 ng/mL) did not significantly change the baseline expression of the prodynorphin gene [29], whereas bullatine A (10 μM) markedly increased the prodynorphin expression by 2.0- and 2.5-fold in the absence and presence of LPS, respectively. In agreement with the in vivo findings in the spinal cord, minocycline completely inhibited bullatine A-induced increase in the prodynorphin expression (*P* < 0.05, by one-way ANOVA followed by post hoc Student–Newman–Keuls tests), without affecting the baseline level in the absence and presence of LPS. However, bullatine A at 10 μM did not significantly affect the prodynorphin gene expression in cultured primary astrocytes originated from the cortex (Fig. 6b) or neurons either from the cortex (Fig. 6b) or from the spinal cord (relative expression value 1.0 ± 0.1 vs. 0.9 ± 0.3).

In order to compare the stimulatory effect of bullatine A and guan-fu base A on the prodynorphin gene expression, cultured primary microglial cells were treated with different concentrations of bullatine A (1 × 10^−6^, 3 × 10^−5^, 1 × 10^−5^, 3 × 10^−5^, and 1 × 10^−4^ M) or of guan-fu base A (1 × 10^−6^, 3 × 10^−5^, 1 × 10^−5^, 3 × 10^−5^, 1 × 10^−4^ and 3 × 10^−4^ M) for 6 h and the cellular prodynorphin gene was measured by real-time PCR. Cell viability was not affected under the tested bullatine A or guan-fu base A. As shown in Fig. 6c, bullatine A increased the prodynorphin gene expression in a concentration-dependent manner, with an EC_50_ value of 3.2 μM. In contrast, guan-fu base A up to 300 μM did not significantly affect the expression of prodynorphin.

Treatment with LPS produced an extraordinary increases (26-, 30-, and 35-fold) in the expressions of TNF-α, IL-1β, and IL-6 mRNA, respectively (*P* < 0.05, by one-way ANOVA followed by post hoc Student–Newman–Keuls tests). As shown in Fig. 6d–f, minocycline completely blocked LPS-induced overexpression of TNF-α, IL-1β, and IL-6 (*P* < 0.05, by one-way ANOVA followed by the post hoc Student–Newman–Keuls tests), although it did not affect their baseline expressions. In contrast, the treatment with bullatine A did not significantly inhibit the gene expression of those pro-inflammatory cytokines in the presence or absence of LPS stimulation.

### Bullatine A induced dynorphin A upregulation in spinal microglia in neuropathic rats

**Fig. 7:**
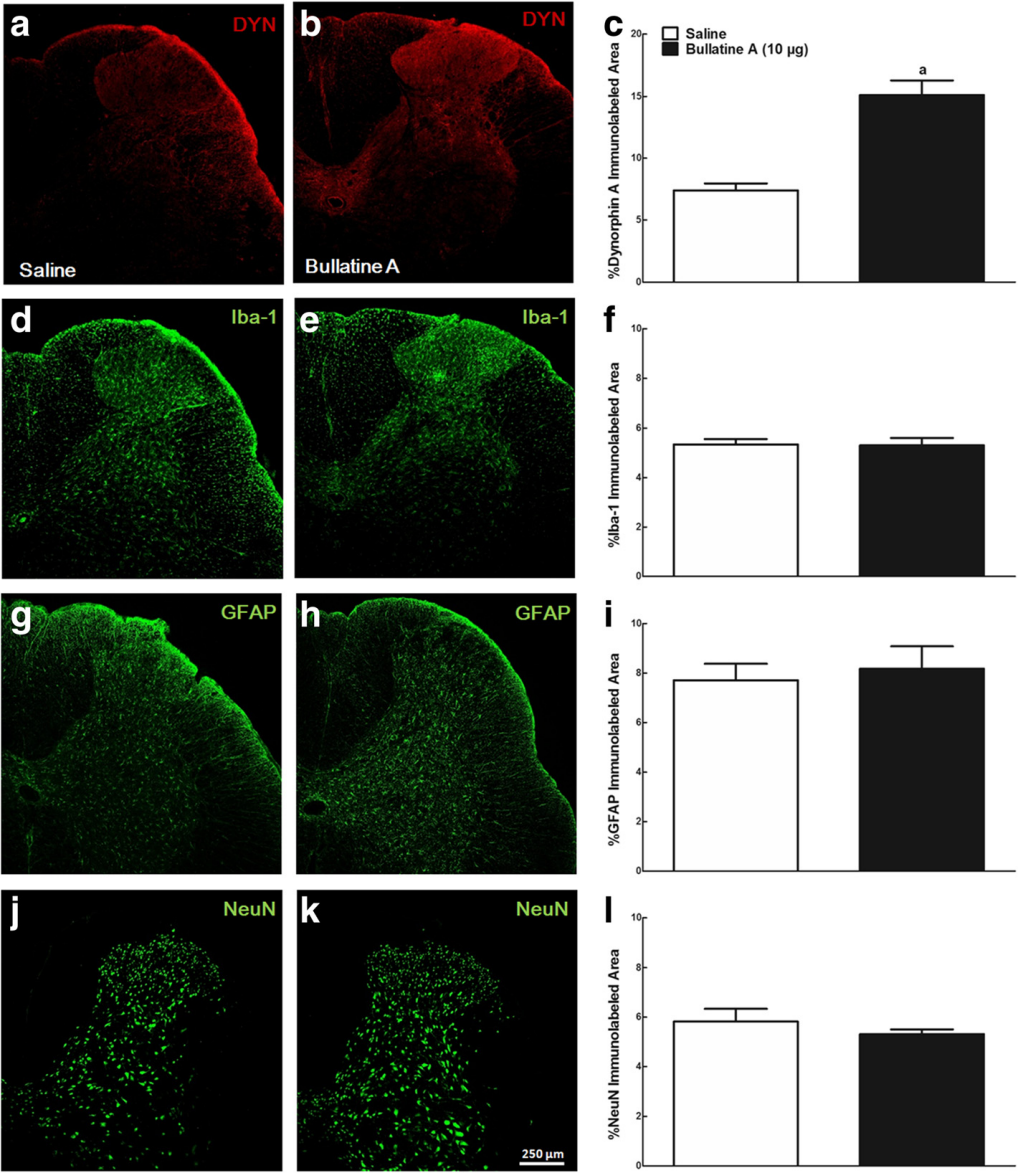
Stimulatory effects of intrathecal injection of bullatine A on dynorphin A immunofluorescence staining in the ipsilateral spinal dorsal horn of neuropathic rats. Peripheral neuropathy was induced by unilateral L5–L6 spinal nerve ligation for 2 weeks, and frozen sections were obtained 1 h after intrathecal saline (10 μL) or bullatine A (10 μg) treatment. Immunofluorescence was labeled with the dynorphin A antibody (**a**, **b**), microglial marker Iba-1 (**d**, **e**), astrocytic marker GFAP (**g**, **h**), and neuronal marker NeuN (**j**, **k**). Photomicrographs were taken from the ipsilateral spinal cords. The immunolabeled surface areas for dynorphin A (**c**), Iba-1 (**f**), GFAP (**i**), and NeuN (**l**) were quantified from the spinal dorsal horn (laminae I–V) using the ImageJ computer program. Data are expressed as mean ± SEM (*n* = 6 in each group). Symbol *a* denotes statistical difference from the spinal cord group treated with saline (*P* < 0.05 by unpaired and two-tailed Student *t* test). *Scale bars*: 250 μm

We examined the differential distribution of dynorphin A in the spinal cord of neuropathic rats treated with intrathecal saline (10 μL) or bullatine A (10 μg). In control rats, the dynorphin A immunofluorescence staining was widely expressed throughout the white and gray matter of the spinal cord, particularly in laminae I–V of the dorsal horn and dorsolateral to the central canal (Fig. 7a), in agreement with previous immunohistochemical staining in naïve and polyarthritic rats [42]. After 1 h treatment of bullatine A, dynorphin A staining was much more intense in the ipsilateral dorsal horn (Fig. 7b). Upregulation of dynorphin A expression in the ipsilateral dorsal horn I–V laminae was quantified using the computer-assisted image quantification program. Compared to saline-treated rats, bullatine A significantly increased the averaged percentage immunolabeled surface area by 104 % (*P* < 0.05, unpaired and two-tailed Student *t* test) (Fig. 7c). Immunofluorescence was also stained with the microglial marker Iba-1, astrocytic marker GFAP, and neuronal marker NeuN. After treating with bullatine A, ipsilateral (laminae I–V) expressions of microglial (Fig. 7d–f), astrocytic (Fig. 7g–i), and neuronal (Fig. 7j–l) markers were not significantly changed, compared to the saline-treated rats.

**Fig. 8:**
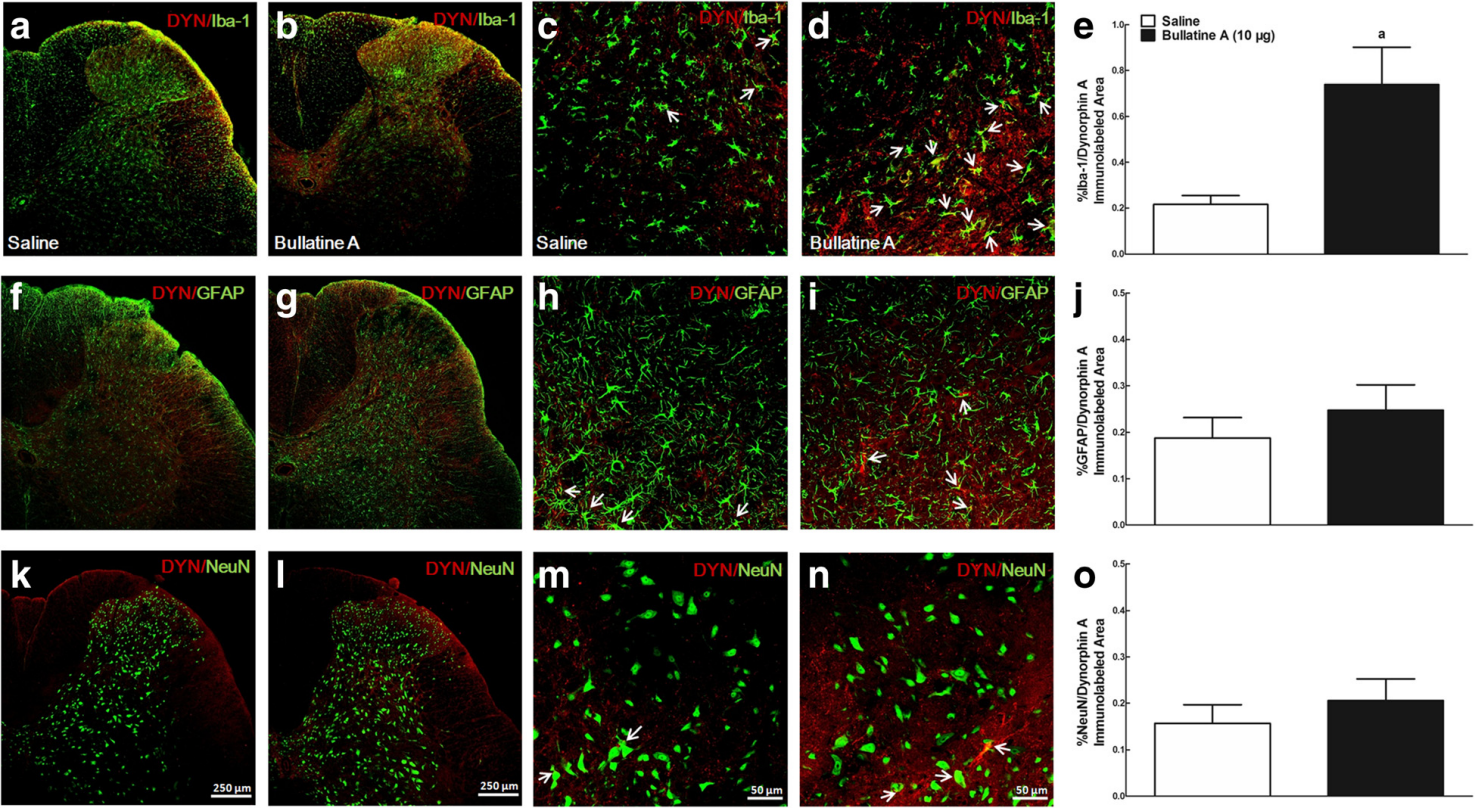
Specific stimulatory effects of intrathecal injection of bullatine A on dynorphin A expression in microglia in the spinal cord using double immunofluorescence staining technique. Peripheral neuropathy was induced by unilateral L5–L6 spinal nerve ligation for 2 weeks, and frozen sections were obtained from the spinal lumbar enlargements 1 h after intrathecal saline (10 μL) or bullatine A (10 μg) injection. The double fluorescence immunostaining was labeled dynorphin A with the microglial marker Iba-1 (**a**–**d**), astrocytic marker GFAP (**f**–**i**), and neuronal marker neuronal nuclei NeuN (**k**–**n**). Photomicrographs were taken from the ipsilateral spinal cords. *Arrows* indicate double-labeling of dynorphin A with cellular biomarkers. The immunolabeled surface areas for double dynorphin A/Iba-1 (**e**), dynorphin A/GFAP (**j**), and dynorphin A/NeuN (**o**) were quantified in the spinal dorsal horn (laminae I–V) using ImageJ computer program. Data are expressed as mean ± SEM (*n* = 6 in each group). Symbol *a* denotes statistical difference compared to the saline control group (*P* < 0.05 by unpaired and two-tailed Student *t* test). *Scale bars*: **a**, **b**, **f**, **g**, **k**, and **l**, 250 μm; **c**, **d**, **h**, **i**, **m**, and **n**, 50 μm

Dynorphin A has been reported to be localized and secreted in neurons, astrocytes, and microglia [17, 43, 44]. To identify cell types that specifically promote expression of dynorphin A in the spinal dorsal horn after bullatine A treatment, we performed double immunofluorescence labeling of dynorphin A with microglial, astrocytic, and neuronal cellular markers. As shown in Fig. 8a–d, dynorphin A immunofluorescence was co-labeled with Iba-1 on microglia in the I–V laminae of the dorsal horn in saline-treated rats. Double immunofluorescence of dynorphin A with Iba-1 was significantly upregulated after treatment of bullatine A by 3.4-fold, as measured using the computer-assisted image analysis program (*P* < 0.05, unpaired and two-tailed Student *t* test) (Fig. 8e). Dynorphin A was also co-labeled with GFAP on astrocytes and NeuN on neurons in the spinal dorsal horn. However, there was no significant difference in co-immunofluorescence staining of dynorphin A and GFAP (Fig. 8f–j) or NeuN (Fig. 8k–o) between saline- and bullatine A-treated rats.

### Intrathecal the microglial inhibitor, dynorphin A antiserum, and k-opioid receptor antagonist suppressed bullatine A-induced anti-nociception

**Fig. 9:**
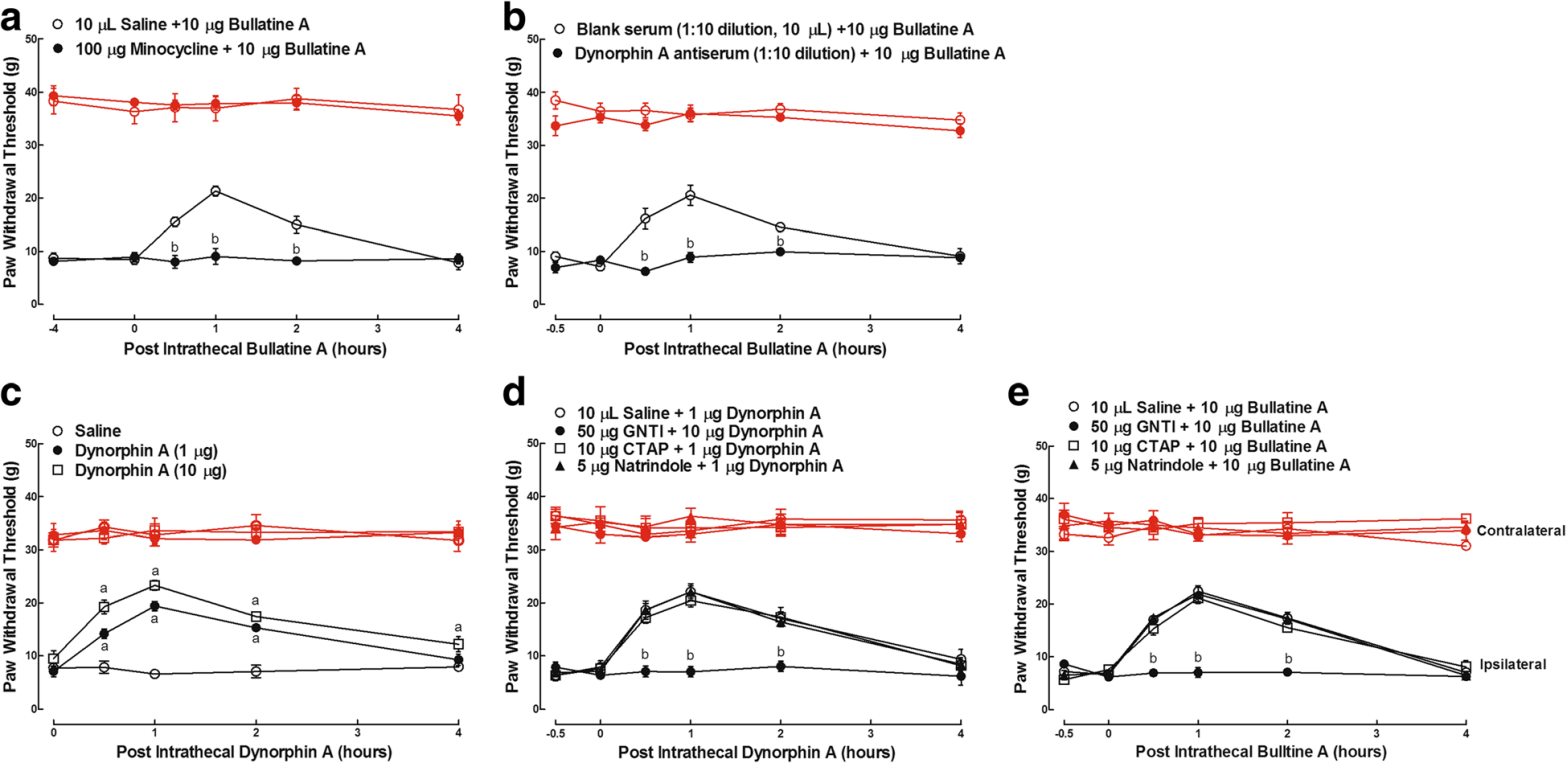
Blockade effects of intrathecal injection of the microglial inhibitor minocycline (**a**), specific dynorphin A antiserum (**b**), and selective k-opioid receptor antagonist GNTI (**c**–**e**) on bullatine A- and dynorphin A-induced anti-allodynia in neuropathic rats. Neuropathic pain was induced by tight ligation of L5/L6 spinal nerves. Minocycline, the antiserum, and opioid receptor antagonists were injected intrathecally 4 and 0.5 h before intrathecal treatment with bullatine A. The data are presented as means ± SEM (*n* = 6 in each group). Symbols *a* and *b* denote statistical significance compared with the saline control group and bullatine A or dynorphin A group (*P* < 0.05, two-way ANOVA followed by the post hoc Student–Newman–Keuls tests)

In order to test whether the spinal microglial dynorphin A expression was causally associated with bullatine A anti-nociception, the microglial inhibitor minocycline, the specific dynorphin A antiserum [17], and antagonists of the opioid receptor subtypes were applied. Two groups of neuropathic rats were first given an intrathecal injection of 10 μL of saline or 100 μg of minocycline, followed by a second intrathecal injection of 10 μg of bullatine A 4 h later. Hindpaw withdrawal thresholds to the mechanical stimulus were measured before and 0.5, 1, 2, and 4 h after the second administration. The effects of minocycline in neuropathic pain are complex when different treatment regimens are applied. It is generally accepted that treatment with minocycline may not be anti-nociceptive in established neuropathy, although its preemptive treatment prevented the induction and/or early development of neuropathic pain [17, 24, 26, 27, 40, 45–48]. As shown in Fig. 9a, intrathecal injection of bullatine A produced time-dependent anti-allodynic effects. Minocycline did not significantly alter the basal withdrawal response in either paws but entirely inhibited the anti-allodynic effects exerted by bullatine A in ipsilateral paws (*P* < 0.05, by two-way ANOVA followed by the post hoc Student–Newman–Keuls tests).

Similarly, two groups of neuropathic rats received intrathecal injection of 10 μL of blank rabbit serum or 10 μL of the dynorphin A antiserum (1:10 dilution). Each rat received a second intrathecal injection of 10 μg of bullatine A 0.5 h later. The paw withdrawal responses to the mechanical stimulus were measured. The time and dose regimen of the dynorphin A antiserum was based on the previous publications [17]. Intrathecal injection of bullatine A produced time-dependent anti-allodynia in the ipsilateral paws. The dynorphin A antiserum entirely prevented bullatine A from suppressing mechanical allodynia (*P* < 0.05, by two-way ANOVA followed by the post hoc Student–Newman–Keuls tests), although it did not significantly alter the basal withdrawal response in either paws (Fig. 9b).

As dynorphin A has variously been reported to inhibit [49, 50] or facilitate pain [51, 52], the effect of dynorphin A on nociception was tested in our conditions. Three groups of neuropathic rats received intrathecal injection of saline (10 μL) and two non-paralytic doses (1 and 10 μg) of the synthetic dynorphin A. Intrathecal administration of dynorphin A(1–17) dose-dependently suppressed mechanical allodynia in ipsilateral paws, with the peak effect (48.8 and 60.5 % MPE) at 1 h and a duration of 4 h (*P* < 0.05, by two-way ANOVA followed by the post hoc Student–Newman–Keuls tests) (Fig. 9c).

In physiological conditions, dynorphin A modulates anti-nociceptive response primarily through the activation of the k-opioid receptor located on neurons in the descending inhibitory system, although it has some affinity for μ- and δ-opioid receptors [53, 54]. To confirm which subtype of opioid receptors was responsible for dynorphin A anti-nociception, we tested the possible blockade effects of the selective μ-opioid receptor antagonist CTAP [55], k-opioid receptor antagonist 5′-guanidinonaltrindole (GNTI) [56], and δ-opioid receptor antagonist naltrindole [57] on dynorphin A-exerted anti-allodynia. It is noted that we selected GNTI instead of the traditionally used nor-binaltorphimine as the latter was recently reported to be an inverse agonist of the k-opioid receptor [58, 59]. Four groups of neuropathic rats received the following pairs of intrathecal injections: saline (10 μL) + dynorphin A(1–17) (1 μg), GNTI (50 μg) + dynorphin A(1–17) (1 μg), CTAP (10 μg) + dynorphin A(1–17) (1 μg), and naltrindole (5 μg) + dynorphin A(1–17) (1 μg). The second treatment occurred 0.5 h after the first injection, and the paw withdrawal responses to the mechanical stimulus were measured before and 0.5, 1, 2, and 4 h thereafter. As shown in Fig. 9d, intrathecal injection of GNTI, but not CTAP or naltrindole, totally prevented the anti-allodynic effect of dynorphin A (*P* < 0.05, by two-way ANOVA followed by the post hoc Student–Newman–Keuls tests), although it was ineffective in altering the basal withdrawal thresholds in both paws.

Lastly, the above selective opioid receptor antagonist was employed to determine which subtype of opioid receptors participated in bullatine A anti-nociception. Four groups of neuropathic rats received intrathecal injections of saline (10 μL), GNTI (50 μg), CTAP (10 μg), and naltrindole (5 μg) before being intrathecally given bullatine A (10 μg). The second administration occurred 0.5 h after the first injection, and the paw withdrawal responses to mechanical stimulus were measured. As shown in Fig. 9e, intrathecal injection of bullatine A induced time-dependent anti-allodynia in ipsilateral paws. In contrast to the negative effects of intrathecal CTAP and naltrindole on bullatine A anti-allodynia, intrathecal injection of GNTI completely inhibited the anti-allodynic effects of bullatine A (*P* < 0.05, by two-way ANOVA followed by the post hoc Student–Newman–Keuls tests), without affecting the baseline withdrawal response in both paws.

## Discussion

Our study, for the first time, demonstrated that bullatine A was effective in blocking spinal nerve ligation- and CFA-induced mechanical allodynia and thermal hyperalgesia and diabetic- and bone cancer-induced mechanical allodynia with efficacies of 45–70 % MPE and ED_50_ values of 0.9–1.9 mg/kg by subcutaneous injection. It is worth noting that bullatine A does not show any inhibitory effects of nociceptive responses stimulated by mechanical or thermal stimuli in normal physiological conditions, suggesting that bullatine A was rather anti-hypersensitive than anti-noxious. Our results indicate that bullatine A is effective in the blockade of pain hypersensitivity, independent of pain models employed, and provide the pharmacological evidence to support its potential use for the treatment of chronic pain.

Emerging evidence indicates that spinal microglia activation and microglia-derived pro-inflammatory cytokines, such as IL-1β, IL-6, and TNF-α, play a crucial role in the pathogenesis of neuropathic pain and other pathological conditions [60, 61]. As expected, we observed that LPS and spinal nerve injury dramatically increased expressions of TNF-α, IL-1β, and IL-6 in cultured primary microglial cells by 26- to 35-fold and in the spinal cord by 6- to 14-fold, which were completely blocked by the microglial inhibitor minocycline. Minocycline is a broad-spectrum antimicrobial tetracycline derivative and is generally considered to be a specific inhibitor of microglia (but not astrocytes [62, 63] or oligodendroglial progenitors [64]), although it was reported to have some direct biological effects in neurons [65, 66]. Minocycline has been extensively used to study the role of microglia in experimental models of brain ischemia [67], traumatic brain injury [68], and peripheral nerve injury-induced neuropathy [17, 24, 26, 27, 34, 40, 45]. In contrast, bullatine A (1–50 μM) was recently reported to decrease ATP-induced overexpression of IL-1β, IL-6, and inducible nitric oxide synthase in mouse BV-2 microglial cells [16]. However, our data do not support the observation either in vitro or in vivo, as bullatine A at 10 μM or 10 μg did not inhibit LPS- or spinal nerve injury-induced overexpression of the pro-inflammatory cytokines in either cultured microglial cells or the spinal cord.

Instead, we revealed that the anti-hypersensitivity of bullatine A was produced by the stimulation of dynorphin A expression in spinal microglia. Dynorphin A is an endogenous opioid neurotransmitter produced in various regions of the brain, including the hypothalamus, the striatum, the hippocampus, and the spinal cord [69–71]. The localization and secretion of dynorphin A have been observed in neurons [72, 73], astrocytes [43], and microglia [17]. The effects of dynorphin A on nociception are bimodal depending on physiological vs. pathological conditions, low vs. high concentrations, and time courses during the development of pain hypersensitivity [17]. At low doses, dynorphin A produces analgesia primarily through activation of k-opioid receptors located on the neurons in the descending inhibitory system [50, 53, 54, 74], while at high concentrations it may induce allodynia and paralysis through the activation of neuronal and microglial *N*-methyl-d-aspartic acid (NMDA) receptors [50, 75–77]. The involvement of spinal microglial-expressed dynorphin A in bullatine A anti-hypersensitivity is supported by the following findings. First, bullatine A stimulated prodynorphin expression in the primary culture of microglia (but not of neurons or astrocytes) and the spinal cord and produced anti-nociception, which were completely blocked by the microglial inhibitor minocycline. In addition, double immunofluorescence staining technology revealed that intrathecal bullatine A-stimulated dynorphin A protein in the ipsilateral spinal dorsal horn was specifically co-localized with microglia (but not neurons or astrocytes). Moreover, the specific anti-dynorphin A serum, which has been found to block the anti-allodynic effects of bulleyaconitine A in neuropathic pain [17], entirely eliminated bullatine A anti-hypersensitivity. Furthermore, the highly selective k-opioid receptor antagonist GNTI at the dose to block the anti-nociceptive effect of the synthetic dynorphin A completely inhibited the anti-hypersensitive effects of bullatine A in neuropathy. In contrast, the μ-opioid receptor antagonist CTAP or δ-opioid receptor antagonist naltrindole was not effective in blocking bullatine A anti-nociception. These results indicate that bullatine A exerts anti-hypersensitivity by specifically stimulating spinal microglia to express and secrete dynorphin A, which then acts on the k-opioid receptors located on the post-microglial synaptic neurons.

We have also recently reported that the C_19_-diterpenoid alkaloids aconitine and bulleyaconitine A produced anti-hypersensitivity entirely via stimulation of spinal microglial dynorphin A expression and secretion which were not dependent on the interaction with neuronal sodium channels, and their anti-hypersensitivity was separated from the neurotoxicity [17]. Taken together, our results indicate that both C_19_- and C_20_-diterpenoid alkaloids exhibit dynorphin A expression properties, regardless of their different carbon skeletal structures. However, although the anti-nociceptive efficacy was nearly the same for both bulleyaconitine A and aconitine, the anti-allodynic potency of bullatine A is nearly 10–32-fold less that of bulleyaconitine A in spinal nerve ligation-induced neuropathic rats (ED_50_: 59.5 μg/kg vs. 1.9 mg/kg for subcutaneous injection and 111.3 ng vs. 1.1 μg for intrathecal injection) [17]. In addition, the potency of bullatine A (EC_50_ 3.2 μM) to stimulate dynorphin A expression in microglia is around 76-fold less than that of bulleyaconitine A (EC_50_ 41.9 nM) [17]. The potencies of anti-hypersensitivity and dynorphin A expression stimulation between bullatine A and bulleyaconitine A are well positively correlated, which further supports our note that stimulation of dynorphin A expression in spinal microglia entirely mediates the anti-hypersensitivity of *Aconitum* and its diterpenoid alkaloids. The findings have made a conceptual advancement, as it was generally accepted that the interaction of *Aconitum* and its alkaloids with neuronal sodium channels were responsible for their analgesia and toxicity [4, 5]. Furthermore, it has been reported that glucagon-like peptide 1 receptor (GLP-1) receptor agonism-induced anti-nociception in pain hypersensitivity through spinal microglial expression of β-endorphin [24, 26]. Our findings of bullatine A and bulleyaconitine A further highlight that spinal microglia exert an anti-nociceptive role, in addition to their known pro-nociceptive functions [9–13]. However, it is worthy to note that activation of microglia may not be required for bullatine A and bulleyaconitine A to induce dynorphin A expression, as both of them stimulated dynorphin A expression by similar degrees in the ipsilateral spinal cord from both sham rats and established neuropathic rats [17] and as bullatine A induced dynorphin A expression in the primary culture of microglia with and without LPS challenge.

Mitogen-activated protein kinases (MAPKs) are a family of evolutionally conserved molecules. After being activated by phosphorylation, they play a critical role in cell signaling, particularly in relation to microglial activation [13, 45, 78]. MAPKs include p38, extracellular signal-regulated kinase (ERK1/2) and c-Jun N-terminal kinase (JNK) [79–81]. Minocycline is likely a non-selective inhibitor of MAPKs, as it has been found to inhibit activation of all of three MAPK subtypes [34, 35, 82–86]. We recently discovered that bulleyaconitine A significantly induced cyclic AMP production and activation of cAMP-dependent protein kinase A (PKA); subsequently, PKA specifically regulated the activity of p38 (but not ERK1/2 or JNK) MAPK and its translocation to the nucleus and then phosphorylated the transcription factor cyclic AMP-response element binding protein (CREB), leading to increased prodynorphin expression and anti-nociception (Li et al., unpublished data, 2016). Given that minocycline completely blocked bulleyaconitine A- and bullatine A-induced spinal microglial dynorphin A expression and anti-nociception, and that both bullatine A and bulleyaconitine A are diterpenoid alkaloids and originated from the genus *Aconitum*, we speculate that bullatine A shares the same mechanism with bulleyaconitine A, by which bullatine A stimulates dynorphin A expression by activation of the microglial cyclic AMP/PKA/p38 MAPK/CREB signaling pathway.

The structure-activity relationships of C_18_- and C_19_-diterpenoid alkaloids of *Aconitum* have been extensively studied [87]. The C_8_-acetyl and C_14_-benzoyl groups were found to be essential for aconitines to stimulate spinal microglial dynorphin A expression and subsequent anti-hypersensitivity, while hydroxylation at C_3_ and C_15_ was also helpful for the actions [17]. In contrast, little information has been reported about the structure-activity relationship for C_20_-diterpenoid alkaloids to produce anti-nociception. There are seven types of C_20_-diterpenoid alkaloids in nature and five types exist in the *Aconitum* species. Bullatine A belongs to the denudatine-type (six-membered ring and a chemical bond between C-20 and C-7), and guan-fu base A belongs to the hetisine-type (the most complicated structure in C_20_-diterpenoid alkaloids with a seven-membered ring and a link between N and C-6), with the significant difference in which the hydroxyl group exists in C_15_ only in bullatine A [3, 88]. The biogenetic relationship studies between C_20_- and C_19_-diterpenoid alkaloids revealed that the bio-transformation of denudatine-type (but not hetisine-type) diterpenoid alkaloids is one of the major pathways to form aconitines, which was specifically regulated by the C_15_-hydroxyl group [3]. Our study demonstrated that the treatment with guan-fu base A up to 300 μM did not stimulate dynorphin A gene expression in cultured microglia and intrathecal guan-fu base A up to 100 μg did produce anti-nociception in neuropathy, whereas bullatine A was markedly positive in the parameters with high EC_50_ of 3.2 μM and ED_50_ of 1.1 μg. The differential activities suggest that the C_15_-hydroxyl group may be required for C_20_-diterpenoid alkaloids to express dynorphin A and subsequently anti-nociception.

## Conclusions

Systemic and intrathecal injection of bullatine A specifically and dose-dependently inhibits pain hypersensitivity in a variety of rat models of pain, including neuropathic pain, bone cancer pain, inflammatory pain, and diabetic neuropathic pain. Bullatine A stimulates dynorphin A expression in the spinal cord and cultured primary microglia in a minocycline-sensitive manner. The spinal anti-allodynic effects of bullatine A are entirely blocked by minocycline, the specific dynorphin A antiserum, and the selective k-opioid receptor antagonist, indicating that bullatine A anti-nociception is entirely mediated by the stimulation of spinal microglial dynorphin A expression. Our results suggest that both C_19_- and C_20_-diterpenoid alkaloids stimulate dynorphin A expression, regardless of their different carbon skeletal structures and further highlight that spinal microglia exert an anti-nociceptive role in addition to their known pro-nociception.

## Abbreviations

ANOVA: Analysis of variance
CFA: Complete Freund’s adjuvant
EC_50_: Half-effective concentration
ED_50_: Half-effective dose
*E*_max_: Maximum effect
GLP-1: Glucagon-like peptide 1 receptor
HPLC: High-performance liquid chromatography
IL-6: Interleukin-6
IL-1β: Interleukin-1β
LPS: Lipopolysaccharides
NMDA: *N*-methyl-d-aspartic acid receptor
TNF-α: Tumor necrosis factor-α
% MPE: % maximal possible effect
